# A Review on Multiplicity in Multi-Material Additive Manufacturing: Process, Capability, Scale, and Structure

**DOI:** 10.3390/ma16155246

**Published:** 2023-07-26

**Authors:** Ayush Verma, Angshuman Kapil, Damjan Klobčar, Abhay Sharma

**Affiliations:** 1 Department of Mechanical Engineering, Netaji Subhas University of Technology, New Delhi 110078, India; ayush.verma.ug20@nsut.ac.in; 2 Department of Materials Engineering, Faculty of Engineering Technology, KU Leuven, Campus De Nayer, 2860 Sint-Katelijne Waver, Belgium; 3 Faculty of Mechanical Engineering, University of Ljubljana, Aškerčeva 6, 1000 Ljubljana, Slovenia; damjan.klobcar@fs.uni-lj.si

**Keywords:** additive manufacturing, multi-material, multiplicity, 3D printing

## Abstract

Additive manufacturing (AM) has experienced exponential growth over the past two decades and now stands on the cusp of a transformative paradigm shift into the realm of multi-functional component manufacturing, known as multi-material AM (MMAM). While progress in MMAM has been more gradual compared to single-material AM, significant strides have been made in exploring the scientific and technological possibilities of this emerging field. Researchers have conducted feasibility studies and investigated various processes for multi-material deposition, encompassing polymeric, metallic, and bio-materials. To facilitate further advancements, this review paper addresses the pressing need for a consolidated document on MMAM that can serve as a comprehensive guide to the state of the art. Previous reviews have tended to focus on specific processes or materials, overlooking the overall picture of MMAM. Thus, this pioneering review endeavors to synthesize the collective knowledge and provide a holistic understanding of the multiplicity of materials and multiscale processes employed in MMAM. The review commences with an analysis of the implications of multiplicity, delving into its advantages, applications, challenges, and issues. Subsequently, it offers a detailed examination of MMAM with respect to processes, materials, capabilities, scales, and structural aspects. Seven standard AM processes and hybrid AM processes are thoroughly scrutinized in the context of their adaptation for MMAM, accompanied by specific examples, merits, and demerits. The scope of the review encompasses material combinations in polymers, composites, metals-ceramics, metal alloys, and biomaterials. Furthermore, it explores MMAM’s capabilities in fabricating bi-metallic structures and functionally/compositionally graded materials, providing insights into various scale and structural aspects. The review culminates by outlining future research directions in MMAM and offering an overall outlook on the vast potential of multiplicity in this field. By presenting a comprehensive and integrated perspective, this paper aims to catalyze further breakthroughs in MMAM, thus propelling the next generation of multi-functional component manufacturing to new heights by capitalizing on the unprecedented possibilities of MMAM.

## 1. Introduction

**Figure 1:**
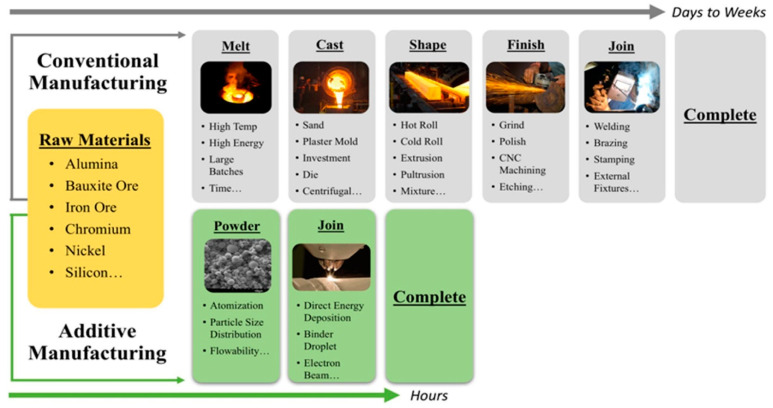
Traditional vs. additive manufacturing [3].

Currently, with the advent of industrialization and market globalization, to meet the requirements of a fast-growing economy, there is a widespread need for an efficient manufacturing process that is reliable, offers less lead time, has greater adaptability of materials, has less waste, requires little or no post-processing, uses inexpensive tools, and is cost-effective in terms of materials and warehouses. While conventional manufacturing processes have been widely used in several domains, they often fail to fulfill all the requirements of today’s products. In the last few decades, additive manufacturing (AM) has filled this gap. The major differentiating factor between conventional manufacturing and AM is the lead time, as shown in Figure 1. Though Figure 1 shows the powder as a feed stock material, the comparative advantage of AM in terms of lead time while using other feedstock material is equally valid. AM, also called 3D printing, rapid prototyping, or solid freeform fabrication, is the layer-by-layer manufacturing of 3D components based on their digitally designed models. These computer-aided design models are converted into standard tessellation language files and then sliced using appropriate software. AM has paved a new way for designing and manufacturing difficult-to-make and complex near-net-shape parts with a minimum cost, a highly automated process, less time, reduced weight through a variety of infill methods, and greater efficiency, which was previously impossible using conventional manufacturing processes. While in the very beginning, AM was seen as a rapid prototyping process only for visualization purposes during the design evaluation phase of mechanical elements, it has become widely accepted as an alternative to traditional manufacturing processes in almost every sector—be it automotive, aerospace, electronics, soft robotics, biomedical, dental, defense, food and packaging, sports and recreation, marine, construction, etc.—for directly fabricating end-use products owing to the superior advantages that it has to offer.

**Figure 2:**
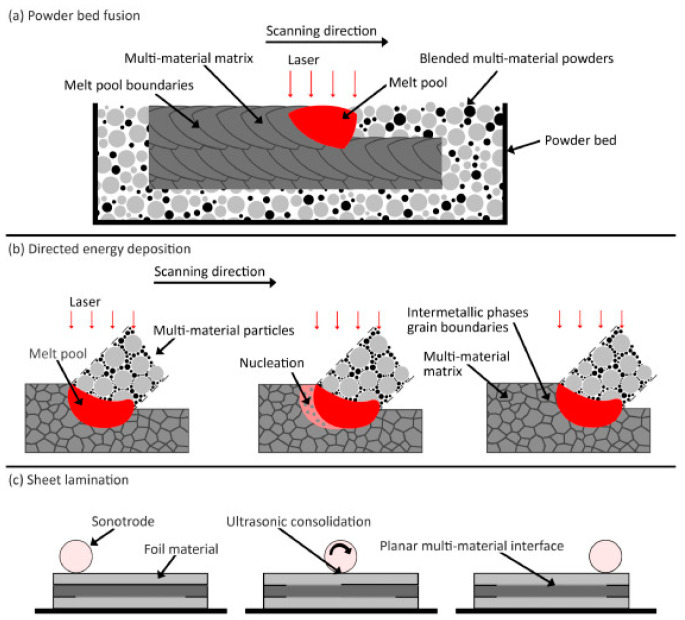
Multi-material metal AM as per ASTM F2792-12a standard [4].

Currently, nearly all the AM processes that have been used are designed to print components using a single material. Current AM systems require improvements in terms of the overall quality of components and their performance for the desired function. Object quality can be addressed by using hybrid AM systems (a combination of additive and subtractive manufacturing processes for better surface finish, accuracy, and precision) or by realizing greater control over the machine, while the performance of the product can be improved by deploying multiple materials in it [1]. Using multiple types of materials during the printing of parts by AM systems is referred to as multi-material additive manufacturing (MMAM). It can impart various properties, namely, mechanical, chemical, electrical, thermal, magnetic, and optical properties, corrosion resistance, fracture toughness, etc., of different materials, such as metals, ceramics, polymers, composites, and resins, to a single component. With the advantage of fabricating multiple materials in a single manufacturing process, it is possible to produce functionally graded materials (FGMs). Functionally graded components are those with varied compositions of material produced intentionally for a specific function. MMAM could deploy certain materials at the specific locations of the component that require their properties the most for a specific function, which increases the efficiency and performance of the component by multifold. Because of these advantages, MMAM has been proven to be an extremely viable option for the fabrication of multifunctional products [2]. With an increasing demand for extending the material portfolio and multi-material components, the AM needs to address the scientific and technological importance pertaining to functional MMAM with controllable properties and create opportunities for new advanced products, e.g., a good strength-to-weight ratio, good wear resistance at particular places, etc. The concept of MMAM is documented in the literature for different processes (Figure 2); however, a technological push is required to create far-reaching impacts (e.g., by validation on industrial case studies), enabling small- and medium-sized enterprises (SMEs) to print multi-material products with in-house designed materials.

The field of AM has substantially evolved over the past decades, and thus, much work and several reviews on MMAM have been presented. Yao et al. [5] proposed a multi-material part design framework that consisted of four stages, namely, (1) the identification of functional and technical requirements, (2) material selection, (3) the selection of the MMAM process, and, finally, (4) deciding on the part geometry and composition of the primary material. In 2013, randomly oriented multi-materials (ROMMs) produced using a PolyJet 3D printing machine were developed by Sugavaneswaran et al. [6]. They concluded that ROMMs with plastic reinforcements showed a higher stiffness than pure elastomeric objects, thus confirming that multi-material AM products show better mechanical properties and hence better functionality in various application areas. In their review of MMAM, Han et al. [7] deduced that MMAM 3D parts can lead to great scientific discoveries and offer geometrically complex multifunctional components. The MMAM limits can be further pushed if software packages that automatically give the best material combination based on the mechanical constraints and bonding capacity, as well as certain algorithms that facilitate the in situ monitoring of the process, are developed. The importance of interface properties for the overall functionality of 3D-printed MMAM parts was investigated by Liu et al. [8], wherein they examined the tensile properties of MMAM-fabricated parts and found that the strength of the parts depends on the interfacial thickness of the primary material and the orientation during printing. Multi-material 3D printing using directed energy deposition (DED) has been critically analyzed by Feenstra et al. [9] in their critical review of MMAM via DED. They completed their review with the conclusion that there are certain issues with multi-material fabrication via DED, such as interfacial cracking due to the insufficient bonding of dissimilar materials; however, DED is still a feasible option for printing multi-material parts using the current technology. Wang et al. [10], in their review on advancements in MMAM via laser powder bed fusion (LPBF), illustrated several issues, such as process simulation issues, contamination during material changes, and pore and crack formation during the LPBF of multi-material structures, and how they can be minimized by a variety of methods, including the proper optimization of printing process parameters. According to Bandyopadhyay et al. [3], a variety of feedback sensors/mechanisms can be implanted in MMAM-printed objects for real-time monitoring and analysis, especially in the medical sector, in the years to come. They further mentioned that MMAM has great potential for fabricating products with new and improved materials, hence combining their superior properties, and it will continue to advance in the near future. MMAM is likely to be used by most manufacturers and designers and even for day-to-day products that we use considering the great merits rendered by it, such as reduced costs and tooling.

The AM of components by employing more than one material, varying the composition of a single material, or using collective processes to fabricate them is termed multiplicity in AM. This multiplicity is often applied to acquire some tailored characteristic properties of numerous materials or processes in a single end-use product for better performance. Multiplicity in the raw material to print MMAM objects is realized in the form of the deposition of multiple materials of the same material type, such as metal–metal or polymer–polymer, during the process or the deposition of multiple materials of different types, such as ceramic–metal or metal–polymer. The combination of two or more 3D printing processes or the use of hybrid additive manufacturing (HAM) processes to build MMAM products is what we call multiplicity in the process. Functional multiplicity comprises fabricating bi-metallic components, creating functionally gradient products, performing in situ alloying, and developing superior composites, i.e., materials that can be used as biomaterials for tissue engineering (which need to be compatible with body tissues and should not produce any toxic substance inside the human body), smart materials, semiconductor materials, etc. Multiplicity can also be seen in the scale of printing MMAM components—for example, combining laser-based DED with wire arc- or electron beam-based DED. Moreover, using multiple processes with various raw materials results in forming varied unique structures, which fall under multiplicity in structure.

Several review papers have been published in the last few years on the multi-material aspects of AM. Most of these review articles focus on the material, process, and application aspects of polymer printing, which include the scientometric analysis and systematic review of the MMAM of polymers by Zheng et al. (2021) [11]; a review of the bonding and strengthening techniques for the PLA biopolymer in MMAM by Brancewicz-Steinmetz and Sawicki (2022) [12]; a review of the capabilities and potential applications of functional materials via vat photopolymerization, examined by Shaukat et al. (2022) [13]; a review of the design for multi-material manufacturing using Polyjet printing by Chadha et al. (2022) [14]; a review of the design of multi-material/multicolor fused deposition modeling 3D printing by Boulaala et al. (2020) [15]; and a review of techniques, properties, and applications by García-Collado et al. (2022) [16]. One of the most cited reviews on additive manufacturing, by Singh et al. [17], focuses on additive bio-manufacturing, excluding other capabilities like FGM/compositionally graded materials (CGMs) and in situ alloying.

Another group of review articles focus on methods and applications—for instance, the reviews by Han and Lee (2020) [7], Yao et al. [18], Zheng et al. (2021) [19], and Rafiee et al. (2020) [20]. A few specific groups of materials have also been reviewed—for instance, construction material was reviewed by Pajonk et al. (2022) [21], cellular metamaterials were reviewed by Nazir et al. (2023) [22], and composite parts were reviewed by Toursangsaraki (2018) [23]. MMAM has been reviewed from the process aspects; for instance, Vaezi et al. (2013) [1] reviewed the processes being used for MMAM, while Hasanov et al. (2021) [2] talked more about the progress and issues associated with different MMAM processes. Among the metal processes, the laser fusion bed has been extensively reviewed in the articles by Wei and Li (2021) [24], Wang et al. (2022) [10], Schneck et al. (2021) [25], and Demir et al. (2022) [26] Wei et al. (2020) provided an overview of laser-based multiple metallic material additive manufacturing, discussing its applications and capabilities at different scales [27]. In addition, Bandyopadhyay and Heer (2018) reviewed the multi-material structures, exploring the materials and processes involved [3]. Recently, Esfarjani et al. (2022) reviewed the topology optimization of additive-manufactured metamaterial structures, specifically examining different types of multi-materials [28], while Bartolomeu and Silva (2022) discussed MMAM for advanced high-tech components, highlighting its importance in producing complex parts [29]. 

Although several reviews have been carried out on additive manufacturing, they are mainly process-specific or material-specific and lack a consolidated view of the process capabilities, scale, and structure. We need to unite them all under a single umbrella of processes and materials in terms of the scale, capability, structure, and application aspects, which we have aimed at in this extensive review paper. Specifically, the aspects of scale, capability, structure, and applications make this review paper different from the available literature.

With this review, we aim to shed some light on the multiplicity in the process, material, capability, scale, and structure in MMAM. The mechanical properties and microstructures of different MMAM-fabricated products are compared with those of conventionally fabricated products, and the variety of materials that can be formed with MMAM systems, such as in situ alloys, FGMs/CGMs, and biomaterials are presented. An attempt has been made to properly study the existing literature to uncover the wide-ranging capabilities of MMAM. The advantages, issues, current and foreseen practical applications, and challenges in MMAM are highlighted. The review concludes with the future scope and research opportunities that are possible in MMAM. The objectives of this review are to update the current knowledge in the field of multiplicity in AM, to foster research growth in MMAM for future developments, and to make MMAM realize its full potential for mass production.

## 2. Methodology

The literature included in the present review has been acquired from online databases. This review was conducted by exploring articles from scientific platforms: Science direct, Scopus, Google Scholar, and Web of Science. Only papers in the English language have been selected. The keywords used for searching relevant articles include, but are not limited to, “additive manufacturing”, “multi-material”, “multimaterial” multi-material additive manufacturing”, “3D printing”, and “functionally graded materials”. Multiple combinations of these keywords were employed to avoid the bias effect. The search procedure records studies of peer-reviewed research papers, proceedings of renowned international conferences, book chapters, and websites pertaining to AM. In the first stage, an in-depth study of the abstracts, the conclusions, and the method sections was conducted to filter the most relevant papers. In the second stage, the results and discussion sections of the papers were analyzed from the perspectives of the process, material, capability, scale, and structure that are relevant to the scope of this review. Note that ISO/ASTM 52900:2021 nomenclature has been adopted throughout the manuscript. In addition, in the succeeding sections, the process names used in the respective articles in the literature have been preserved.

### Structure and Scope of the Review

While this review focuses on the multiplicity aspects of MMAM, it is important to state the scope and structure of the review. Section 3 discusses the implications of multiplicity in terms of its advantages, applications, challenges, and issues. Section 4 provides a detailed review of the multiplicity in MMAM in terms of the processes, materials, capabilities, scale, and structure, respectively. More specifically, Section 4.1 introduces the various AM processes, viz., DED, material extrusion, vat photopolymerization, binder jetting, material jetting, sheet lamination, powder bed fusion (PBF), and hybrid AM. The adaptation of each of these processes for MMAM is discussed with specific examples from the literature. The specific merits and demerits and innovative applications of these processes for MMAM are also addressed in this section. Section 4.2 provides a detailed review of the materials in MMAM. In terms of the materials aspect, this review covers polymer- and composite-based MMAM, metal–ceramic-based MMAM, metal alloy-based MMAM, and biomaterials. Each of the material classes is discussed and supported by relevant examples from the literature. Section 4.3 then provides the wide-ranging capabilities of MMAM in terms of its ability to fabricate bi-metallic structures and functionally/compositionally graded materials. The flexibility that MMAM provides for designing new and exotic alloys is then discussed. Section 4.4 then provides an overview of the various scales at which MMAM operates, followed by Section 4.5, which discusses the resulting structural changes originating at various scales of operation in MMAM. Section 5 concludes the review, provides future research directions in MMAM, and provides an overall outlook of MMAM, pertaining to its inherent associated multiplicity. 

## 3. Implications of Multiplicity in AM

The onset of AM in recent decades has marked a new era of more economical, reliable, and efficient fabrication methods compared to traditional methods. However, single-material AM cannot always fulfill the requirements of the product target functionality compared with MMAM. The number of advantages that MMAM offers in terms of freedom for the designer, less lead time, etc. has enabled today’s manufacturing businesses to cope with the exponentially increasing international competition in sectors such as automotive, medical (tissue engineering), aerospace, defense, and electronics (3D embedded circuits) sectors. Nevertheless, combining multiple materials such as polymers, ceramics, metals, and alloys or multiple processes simultaneously cannot always be reliable. The bonding of dissimilar materials, the lack of an ability to operate them with similar conditions and equipment, the need for postprocessing, and poor dimensional accuracy and size are still some great challenges for the MMAM community to overcome. The issue of post-processing in MMAM has been addressed in some of the recent publications [30]. However, the residual stress and distortion issue due to thermal mismatching in MMAM still requires great attention; thermal management has successfully managed this in single-material AM [31]. MMAM has been used and tested by a number of manufacturers and researchers across the world because issues such as a lack of interfacial bonding can greatly affect the overall reliability and trustworthiness of MMAM-produced goods. Thus, keeping this in mind, we discuss some of the advantages, issues, and challenges of MMAM processes based on the work that has been carried out in the past.

### 3.1. Advantages

3D printing processes, especially those using multi-materials, offer numerous merits compared to conventional or single-material AM processes. Some of them include the great advantage that they give to the designers in terms of multiple feedstock options, allowing the designer to incorporate special materials at special locations, thus creating multifunctional high-performing compact objects with embedded electronic circuitry for complex applications on par with conventionally produced components at a low price and with a shorter built time. Additionally, the lack of the need to assemble various components is another merit that MMAM offers. A product of various parts is built in a single step with MMAM, thus reducing the costs of assembly (nut bolts, rivets, fasteners, etc.). The number of energy sources MMAM uses is minimal compared with that of the traditional multistep methods. MMAM also finishes the desired tasks with minimum waste of the materials, thereby reducing the costs and saving resources, hence acting as a sustainable manufacturing method [1]. Several industries, such as aerospace, biomedical, automotive, electronics, and robotics sectors, have utilized MMAM processes considering the merits that MMAM processes guarantee. Gibson et al. [32], in their book on AM, discussed the extensive reliance of a number of industries on MMAM, such as the aerospace industry making use of MMAM processes to build lightweight heat shields composed of multi-material ceramics and highly capable composites for space shuttles. They also discussed the wide use of MMAM by the medical sector for bone tissue engineering and the manufacturing of biocompatible alloys using MMAM for implants. Zhou et al. [33] also emphasized the need to build multi-material 3D printers after they developed a mask-image projection-based vat photopolymerization process incorporating digital materials into it, which can be used to develop 3D objects with multiple functionalities and wide applications due to the excellent combination of mechanical, electrical, chemical, thermal, biological, and optical behaviors of several materials. Thus, MMAM has the potential to become the most sought-after fabrication method at present.

### 3.2. Applications

**Figure 3:**
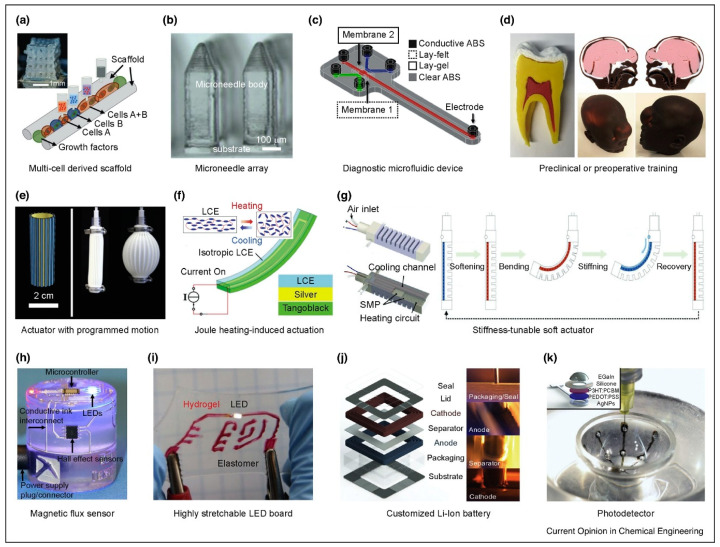
Overview of applications of MMAM: (**a**–**d**) biomedical engineering, (**e**–**g**) soft robotics, and (**h**–**k**) electronics [7].

Single-material AM systems cannot meet the needs of applications requiring multi-material production from a single machine, e.g., 3D circuits, embedded components, and medically compatible implants. Multiplicity in MMAM provides a one-stop solution that can be utilized in numerous actual industrial applications. The automotive industry employs multi-material design (components with compliant hinges, taillights with multiple colors, etc.), to which MMAM can contribute to a great extent. In the aerospace sector, MMAM can be utilized to produce optimally designed lightweight structures that can enhance performance and reduce costs. One particular potential application could be the production of multi-material heat shields for space shuttles [32]. MMAM has already been employed in the medical sector to produce biocompatible implants. MMAM provides the ability to produce implants that contain a strong material in the core, surrounded by a material compatible with the bone tissue, along with a low-friction material in the joint area. In tissue engineering, MMAM could provide the ability to print artificial replacement organs on demand [1]. MMAM can alleviate the limitations faced by scaffold-based tissue engineering with the adaptation of innovative approaches such as organ bioprinting [34], the laser writing of cells [35], bio electrospraying [36], and biological laser printing [37]. The production of hybrid scaffolds that show much better mechanical properties than those produced with hydrogels has been reported [38]. Other applications of MMAM in the medical field include the fabrication of biohybrid cantilevers and actuators with hydrogels and cardiac cells [39], the precise insertion of cells and proteins at desired locations of the structure, the changing of the synthetic material composition, etc. Multi-material stereolithography has a wide range of applications in microelectromechanical system (MEMS) technology and the production of micro-optics and microchemical devices for BioMEMS [40]. Multiplicity in the process in MMAM allows for the rapid manufacturing of multi-material embedded systems. Figure 3 provides an overview of many real-life applications enabled by MMAM.

### 3.3. Challenges and Issues

The proper joining of dissimilar materials such as polymers–metals and metals–ceramics has been one of the greatest challenges of MMAM. Different materials have different thermal behaviors, coefficients of thermal expansion, melting and boiling points, chemical structures, and solidification rates. Thus, combining them in similar environments with similar constraints often poses great challenges to manufacturers and designers. The joining of dissimilar materials has greatly benefited from the development of innovative processes such as magnetic pulse welding [41], impact welding [42], and friction stirring [43]; however, the use of such innovations has limited success in AM and MMAM as well. The use of adhesives to bind the two bonded materials is mentioned as an alternative; however, using a third material increases the overall costs and weight and entails a new process, which is not feasible for every designer and industry. Lumpe et al. [44] examined the tensile strength of MMAM interfaces built by the material jetting process. They found that both the process parameters and the material combination strategies determine the tensile behavior of the printed part. Newer exotic material combinations and interface adjustments can adversely affect the interface integrity. Vu et al. [45] studied the integrity of the interfacial bonding of MMAM-printed objects, and the results indicated that most of the cracks/fractures that further lead to fatigue failure tend to occur in the interfacial zone. Hasanov et al. [46] examined the strength of FGMs printed by the fused filament fabrication (FFF) process. They found that the quality or reliability of the joined product depends on the interlocking capacity between the dissimilar polymer chains. Thus, properly characterizing thermal and mechanical properties at the interface is crucial for a sound MMAM process. 

Other challenges include the possible contamination of materials during material changes, which is a serious concern, and the lack of a proper, robust computer design system with a preloaded database of materials that is equipped to process multi-material pre-slicing so that a calculation of the placement/optimal distribution of materials, which is very complex, can be performed based on the desired functionality and application during the design phase itself, keeping in mind the dissimilar characteristics of the materials to be combined, without actually building the prototype [1]. New extruder fabrications or designs can address challenges such as the improper mixing of multiple materials during printing in widely used processes such as FFF [2]. Hence, further research on these challenges will exponentially increase the use of MMAM, considering the immense capabilities of AM processes, particularly those using multi-materials. 

## 4. Multiplicity in MMAM

### 4.1. Processes Used for MMAM

With the growing need for complex geometries and difficult-to-build products, AM has evolved as a solution for manufacturers by offering less lead time, better products, reduced costs, and less waste. However, most AM systems employ single materials to print components, thus restricting the functionality of the developed products. With the advent of MMAM, which produces superior products with multiple materials such as polymers, ceramics, metals, and alloys, either many AM processes have been modified to employ multiple materials during printing or two or more AM processes have been combined to facilitate MMAM. Different processes have their merits and specific demerits, such as the inability to use multiple or certain types of materials, a lower dimensional precision, and a poor surface finish. AM processes are broadly classified into seven types, namely, powder bed fusion (PBF), DED, vat photopolymerization, material extrusion, material jetting, sheet lamination (laminated object manufacturing (LOM) and ultrasonic consolidation (UC)), and binder jetting. HAM processes combine additive and subtractive manufacturing methods for a better surface finish and superior advantages [2]. A brief overview of various AM processes, technologies that fall under them, and downsides can be seen in Table 1. 

#### 4.1.1. Directed Energy Deposition (DED)

**Figure 4:**
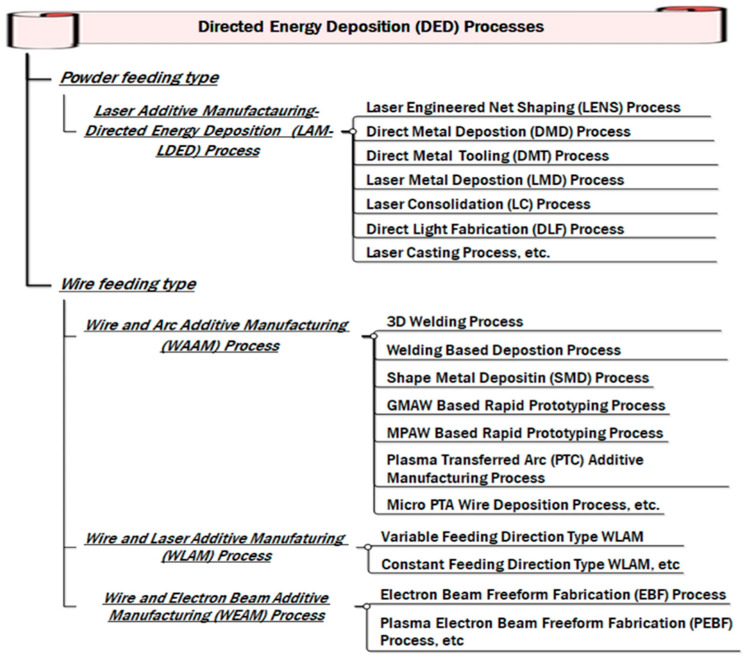
Classification of DED processes [48].

The DED process involves using raw materials as wires or powders directly deposited on the substrate by melting them in a controlled heated region using direct focused energy in the form of a laser, electron beam, or plasma arc. An inert gas is also used to protect the molten pool from contamination [2]. An extensive classification of DED processes is shown in Figure 4. Heterogenous components with tailored characteristics can be formed with DED, which deposits materials by melting them in a line-by-line fashion on the substrate, after which they solidify.

**Figure 5:**
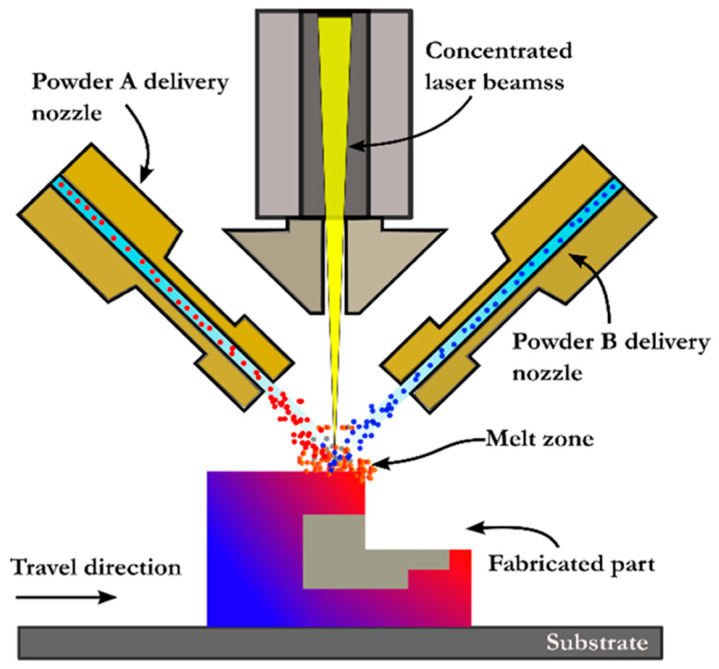
Schematic diagram of the DED process using powder feedstock and laser power [2].

Due to their numerous advantages, such as being able to change powders at any time during the process, use premixed powders without disrupting the process, and change the composition of the material being deposited to produce FGM structures with greater functionality, DED processes have been extensively utilized by manufacturers and researchers across the globe to build multifunctional products [2]. A schematic diagram of the laser-based DED process using powder feedstock is shown in Figure 5. In 2010, Zheng et al. [49] used the LENS (DED process using a laser energy source) to deposit Ni-coated and uncoated TiC reinforcement particles on an IN625 Ni-based MMC. After proper analysis and characterization, the resulting component showed a much higher strength than a single alloy. Similarly, Bandyopadhyay et al. [50] used laser processing to add calcium phosphates to CP-Ti or Ti6Al4V alloys to increase its wear properties by forming a tribofilm that acts as a solid lubricant. Thus, the formed composites can be used to increase the life of titanium implants in joint replacements. Laser-based DED processes were also used to fabricate lead zirconate titanate (PZT) structures on a metallic substrate via melting and solidification by Bernard et al. [51]. The PZT structures imparted good dielectric properties without needing further heat processing and could thus be used for making embedded sensors. Some demerits of DED-produced MMAM products include the extensive use of traditional materials instead of materials specifically tailored for DED, the development of thermal stresses, post-processing requirements for a better surface finish, intermetallics, cracking, etc.

#### 4.1.2. Material Extrusion Process

**Figure 6:**
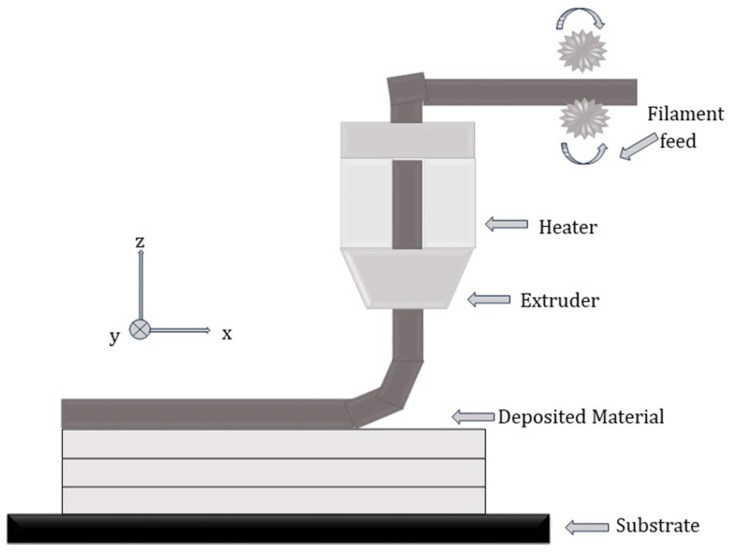
Material extrusion (adapted from [54]).

Extrusion-based 3D printing is a widely used technique due to its various advantages, such as the fabrication speed, a wide variety of materials (polymers, ceramics, and composites), and a low waste of feedstock [1]. Material extrusion is used to fabricate a structure by depositing the raw material in a line-by-line, layer-by-layer fashion on the base substrate after the material is melted inside the extrusion head (Figure 6). Extrusion-based AM techniques can be divided based on whether the material is deposited with or without melting. Fused deposition modeling (FDM) is based on extrusion using melting methods, whereas low-temperature deposition modeling (LDM) falls under extrusion processes without melting. Two or more deposition nozzles are used in these types of processes to facilitate the use of multi-materials while printing. Khondoker et al. [52] used two polymers immiscible with each other to print FGMs using a multi-material FDM process. The objective was to improve the bonding between thermoplastics without using external chemicals. The resulting component showed no adhesion failure, thus giving the material mechanical strength.

Extrusion-based systems have been excessively used in the biomedical sector because the process is convenient to use with biomaterials. Xiong et al. [53] used low-temperature deposition modeling to manufacture poly(l-lactic acid) and tricalcium phosphate composites for tissue engineering. Compared with other AM processes, the LDM process proved to be a better manufacturing method because it protects the bioactivity of the tissue materials due to its non-melting liquifying method of depositing materials. The major downsides of extrusion-based methods include a lower accuracy, lower mechanical strength, and poor interfacial bonding capacity.

#### 4.1.3. Vat Photopolymerization Process

**Figure 7:**
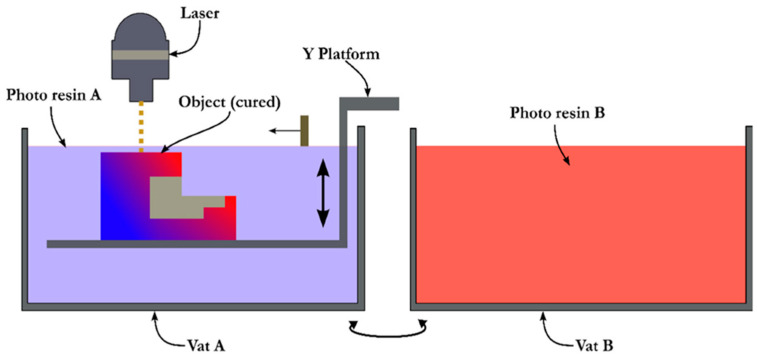
Schematic diagram of the vat photopolymerization process [2].

Vat photopolymerization, as shown in Figure 7, is a process that utilizes ultraviolet light or any light source to fabricate multifunctional products from photopolymer resins. This technology consists of direct light processing and stereolithography (SLA) processes. As soon as the light rays impinge on the deposited resins, solidification occurs due to internal polymeric chain formation. Once a layer has been solidified, the platform carrying the substrate moves down. A new layer of photosensitive liquid-state resin accumulates on the substrate to be cured by light. The material is changed with the help of a rotatable platform containing both resins. Thus, a fully dense structure with multiple materials is obtained. Several advantages of the SLA process, such as highly accurate dimensions while printing and the ability to use transparent materials (due to which living cells can be used throughout them), that are difficult to realize with other AM processes have made vat photopolymerization a popular choice among manufacturers.

Maruo et al. [40] constructed a hybrid component of multiple photopolymer resins using a micro-stereolithography process. The main objective was to combine the superior properties of these resins, such as electrical, optical, and magnetic properties, into a single structure. These structures can be extensively used in manufacturing sensing and actuating devices. Liu et al. [55] also utilized the photopolymerization process in multiple rounds to manufacture three-dimensional hydrogel structures in which living cells are present at a very small resolution. The major drawbacks of using this process include that only photopolymers can be used, waste and feedstock contamination occur while changing it, and changing the raw materials takes excessive time [1].

#### 4.1.4. Binder Jetting Process

**Figure 8:**
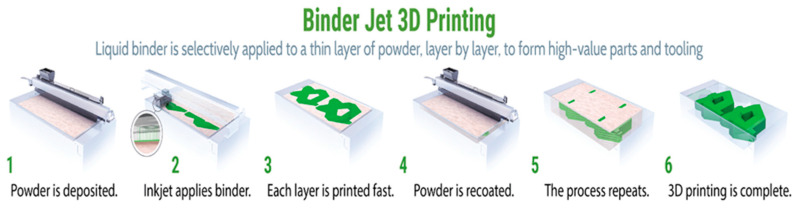
Binder jetting process [58].

The binder jetting (BJ) process involves printing 3D structures by depositing the materials through one or more nozzles, usually using a binder phase to adhere the material together, thus improving the adhesion between the layers. BJ is an AM process that involves no melting but employs sintering to provide functional strength to the part. Inkjet technology is the main binder jetting technology used in 3D printing and was developed at the Massachusetts Institute of Technology, Boston. A few droplets of the binding material are placed onto the powder surface to bind the particles together; the powder bed is lowered, and the same process is repeated until the desired structure is built [1]. Beaman et al. [56] formulated a two-binder configuration for AM. One binder is the normal raw material, while the other contains carbon. Using this system, they aimed to alter the carbon composition in steel parts to obtain the desired variation in strength. The merits of using the BJ process are the fast processing and the various types of materials it can employ. Still, the poor surface quality and high porosity currently often restrict its usage. The typical BJ process is shown in Figure 8. Guessasma et al. [57] tested the quality of droplet-based additively manufactured composites consisting of acrylonitrile butadiene styrene and thermoplastic polyurethane by tensile strength testing and 3D image processing to check the porosity, which deeply controls the mechanical response of the composite.

#### 4.1.5. Material Jetting Process

**Figure 9:**
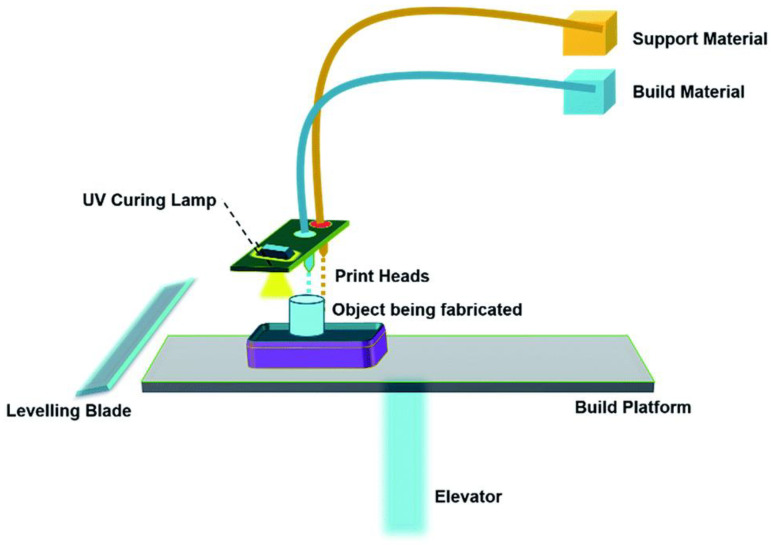
Material jetting process [64].

Material jetting (MJ) is similar in system configuration to binder jetting, as shown in Figure 9, but the basic difference between the MJ process and the BJ process is that the BJ process deposits liquid droplets of adhesive materials onto the powder bed to bind the subsequent powder layers together, whereas, in the MJ process, an inkjet-type print head (with types varying from drop-on-demand (DoD) to continuous inkjet) directly deposits a liquid light-curable polymer resin onto the substrate, which is then solidified by light curing or solvent evaporation. The main drawbacks of the MJ process are the expensive nature of the raw materials, the long processing, the requirement of support material, and the clogging problem in nozzles. At the same time, the advantages include an excellent surface finish, high precision, and less material waste [59]. Large structures that are otherwise difficult to print can be built in the MJ process by incorporating one or two print heads. For example, Hou et al. [60] used DoD-type printing using two print heads, one for the built part material and the other for the support structure material, which gave an overall shape and structure to the printed object. Mott and Evans. [61] printed a functionally graded material based on zirconia-alumina using DoD-type inkjet printing. The obtained FGM profile was continuous, with a finely distributed grain structure like the predicted profile. A multi-material DoD-type inkjet printing configuration was manufactured by Xie et al. [62] using a pneumatic diaphragm-based actuator that can produce thousands of small droplets of materials such as polymers, metals, and optically active materials from the nozzle. Ibrahim et al. [63] configured and altered an inkjet printer to fabricate multi-material 3D structures layer-by-layer. Gradient material interfaces could be fabricated by the MJ process, as proven by Vu et al. [45], who processed elastomeric (TangoBlackPlus) and acrylic (VeroWhitePlus) raw materials for fracture testing.

#### 4.1.6. Sheet Lamination Process

**Figure 10:**
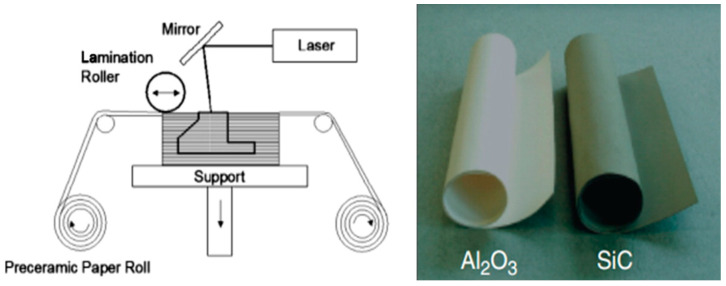
LOM process; preceramic papers of Al_2_O_3_ and SiC [1].

Sheet lamination is a manufacturing technique in which metal, plastic, or any other material sheets are cut, joined, or stacked together to form a component. It is classified into two types of processes based on the joining process utilized to bond the material sheets together; if a binder or a glue is used, then it is termed LOM, and when the sheets are joined by ultrasonic wave joining, it is termed ultrasonic consolidation (UC) [1]. A schematic diagram of the LOM process is shown in Figure 10. UC is a hybrid AM process that uses a milling tool for surface finishing; hence, it is discussed in subsequent sections. Travitzky et al. [65] used the LOM process (Figure 10) to fabricate multi-ceramic structures using preceramic papers, as they can be formed into complex shapes and sizes using this technique. The control of the laser power to prevent sheet damage and the delamination of the material sheets are major issues with LOM [1]. In 2009, Gomes et al. [66] developed green tapes of Li_2_O–ZrO_2_–SiO_2_–Al_2_O_3_ (LZSA) parent glass using the LOM process. Weisensel et al. [67] fabricated carbon templates of laminates from pyrolyzed filter paper sheets using LOM. They deduced that the density and porosity, on which the mechanical response of the structures depends, can be modified by altering the process parameters of the LOM process. The advantages of the sheet lamination process include fast processing and fewer defects, whereas the shrinkage and waste of feedstock are some shortcomings of this process.

#### 4.1.7. Powder Bed Fusion (PBF) Process

**Figure 11:**
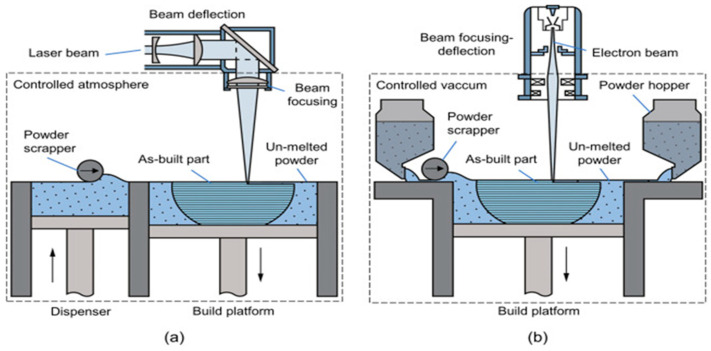
(**a**) LPBF process; (**b**) EBPBF process [59]. The down arrows in the figures represent the direction of movement of the build platform, while the up arrow represents the direction of motion of the dispenser.

**Figure 12:**
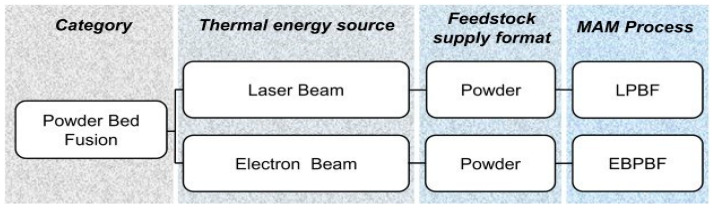
Classification of PBF processes [59].

PBF is an immensely popular AM process, as it offers great material versatility (polymers, ceramics, or metals) and prints components with a considerable strength-to-weight ratio. In contrast to binder jetting, PBF involves the action of a focused thermal energy source to melt the powder feedstock to form a component in a layer-by-layer manner. Selective laser sintering (SLS) and selective laser melting (SLM) are the two processes falling under PBF that are differentiated from each other based on whether there is partial melting or complete material of the raw material. Laser and electronic beams can provide the necessary thermal energy during PBF, further differentiating PBF into LPBF and electron beam powder bed fusion (EBPBF) (schematic diagram illustrated in Figure 11). The classification of PBF processes is shown in Figure 12. In 2003, Lappo et al. [68] fabricated simple geometry tubes using a multi-material SLS process for casting. The PBF process has disadvantages such as a lower accuracy due to the particle size, the reuse of the powder, and the requirement of a special atmosphere during printing [1]. Regenfuss et al. [69] developed a novel approach called laser micro sintering based on the SLS process for manufacturing micro parts using raw materials (metals or ceramics) in the form of powders.

Bi-metallic structures consisting of 316L steel and CuSn10 bronze were developed by Chen et al. [70] using a multi-material SLM process. The effects of input parameters, such as the laser speed and power, on the mechanical behavior and interface properties were examined. Metal-ceramic multi-material structures have also been synthesized using the SLM process. Wang et al. [71] fabricated TiB2/Ti6Al4V multi-materials using the PBF process. Variations in the hardness of the material were observed at the interface. Zhang et al. [72] manufactured metal-glass multi-materials by combining 316L steel and soda lime using a point-by-point powder delivery-based PBF process. Several metal–glass objects can be printed using this novel approach. Laser-based powder bed fusion has also been utilized in the fabrication of metal–polymer-based multi-material structures by Chueh et al. [73]. They printed hybrid metal (Cu10Sn), SS316L, and polymer (PA11) objects using LPBF-based systems. They concluded that the surface quality of Cu10Sn was greatly improved by incorporating PA11 polymer into it.

#### 4.1.8. Hybrid Additive Manufacturing (HAM) Processes 

**Figure 13:**
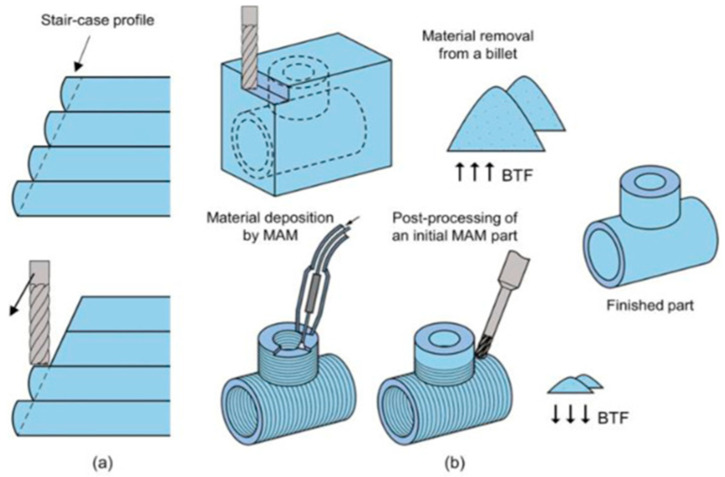
(**a**) Representation of metal removal processes at the post-processing level, and (**b**) comparison of BTF ratios in conventional and HAM machining methods [74].

An HAM process refers to the combination of AM processes such as DED and subtractive/traditional manufacturing processes such as CNC milling and grinding for improving the surface quality, eliminating the demerits of AM processes in terms of the part quality and performance, and making smart use of the merits offered by both types of processes. HAM processes generally combine a cost-effective AM process with a dimensionally precise conventional process. Subtractive processes involve the cutting and removal of materials, thus leading to extensive waste. However, using an HAM process ensures minimum waste of the materials and the maintenance of a good surface finish, as the AM process is first used to produce a near-net-shape component, and then conventional manufacturing methods are applied to obtain a better surface quality [2]. A lower buy-to-fly (BTF) ratio is observed in HAM, while a higher BTF ratio is seen in conventional fabrication methods. The BTF ratio is the ratio of the total volume of the raw material taken to the total volume of the finished product. It signifies the amount of waste of raw material in a manufacturing method. Thus, for a manufacturing strategy to be superior in terms of efficiency and economy, the BTF ratio should be as low as possible. A diagram explaining this notion is shown in Figure 13 [74].

Several researchers worldwide have already carried out much work exploring HAM processes to manufacture better-performing economical components. Bambach et al. [75] used a combination of wire arc additive manufacturing (WAAM) (DED process using wire feedstock) and a metal forming process to fabricate objects from Ti6Al4V titanium alloy. They concluded that using HAM processes resulted in a high material yield and a better performance and flexibility than those of traditional forging methods. Dugar et al. [76] used HAM, combining the WAAM in robotic machining to sustainably produce AiSi5 alloy turbine blades. They deduced that HAM enables an economically viable, resource/energy-feasible, and time-efficient production of complex parts. Pragana et al. [77] studied the process of coin minting using AM processes and then forming. The LPBF process is used to produce coin cylinders. Then, individual finished coins are obtained using the electric discharge machining (EDM) process, and then the polishing and minting of the coins are carried out using appropriate pressing tools. After a series of numerical and experimental tests, they confirmed the efficiency and excellent performance of the hybrid additively manufactured coins. Hybrid AM processes were also utilized by Kapil et al. [78], using a hybrid laser-arc-based directed energy deposition process. Bai et al. [79] experimented with manufacturing 6511 martensitic stainless steel using SLM and milling processes. The effects of input factors on the properties of the obtained specimens, such as the surface residual stress and distribution affected by the milling process factors, were studied. The shape deposition manufacturing (SDM) process is a hybrid AM process that deposits molten materials on a substrate and then uses material removal processes to chip off excess material. Cooper et al. [80] used SDM and CNC milling to form multi-material polymer–ceramic parts. More recently, Farias et al. [81,82] utilized an arc-based DED process integrated with in situ interlayer hot forging and post-deposition heat treatments to significantly refine the typical coarse and highly oriented microstructure of Ni-based superalloy 625. It was observed that the in situ hot forging induced abundant crystal defects that led to the promotion of static recrystallization during post-deposition heat treatments. Li et al. [83], in their work, developed a laminated composite of IN718-SS 316 L using hybrid additive and subtractive manufacturing (HASM). The experimental results suggested that the developed laminated composite had a better performance than single materials of identical sizes. The advantages of HAM are innumerable, including less waste, excellent surface properties, and cost-effectiveness; however, issues such as an increased lead time for machinery changes, a large amount of information to consider before the design, and limitations in feature size due to the geometry of the cutting tool are some of the demerits of using HAM-based fabrication techniques.

The mentioned AM processes serve distinct applications and cannot be substituted by other versions. Consequently, the strengths and weaknesses of these AM processes are also reflected in their respective MMAM applications. It is important to note that the strengths and weaknesses are both material-specific and application-specific. However, in certain cases where multiple processes are feasible, the selection of a specific process can significantly impact the outcome. For instance, comparing the Powder Bed Fusion (PBF) and Directed Energy Deposition (DED) processes, PBF results in a finer interface between two materials than DED. However, DED’s high energy input can lead to thermo-mechanical and material compatibility issues. On the other hand, the DED process provides added strength through its combing action, i.e., interlocking the two materials with its saw-shaped interface. Additionally, DED processes excel in in situ alloying due to their larger melt pool. Moreover, arc-based DED with multiple wires is easier to operate compared to multiple feedstock powder streams. The choice of process is heavily influenced by the specific application. For example, among the five AM technologies (powder bed fusion, directed energy deposition, sheet lamination, binder jetting, and material extrusion), extrusion-based MMAM has shown the greatest potential for multi-functional metallic biomaterials [4]. Similarly, in in situ alloying, DED and PBF share some common and uncommon features, and the selection depends on factors such as the size, scale, and material, as explained later in Section 4.3. As MMAM is an evolving field, materials can be individually deposited using specific processes. This has led to the use of multiple process combinations for depositing particular materials within the MMAM framework. In the future, more studies are expected to address the question of which (multi-)processes are suitable for the specific material combinations used in MMAM.

### 4.2. Materials in MMAM

Combining multiple materials in the manufacturing processes bestows the user or the manufacturer with numerous merits that boost the component’s overall functionality, durability, stability, efficiency, and performance. Some of the most popularly used raw materials consist of polymers (thermosets, thermoplastics, fiberglass, carbon fiber-reinforced polymers), composite materials, metals and their alloys, and metal-ceramics (for better stiffness, etc.), which are used by several manufacturing industries ranging from the healthcare industry to the automotive, biomedical, aerospace, food and packaging, marine, defense, and recreation industries. Popov et al. [84] reviewed critical raw materials (CRMs) used for additive manufacturing. Their review provided in-depth insights into CRM-containing materials processed by AM techniques and outlined the potential for the efficient utilization of CRMs and a reduced dependence on CRMs through the wider industrial usage of AM. 

#### 4.2.1. Polymer- and Composite-Based MMAM

**Figure 14:**
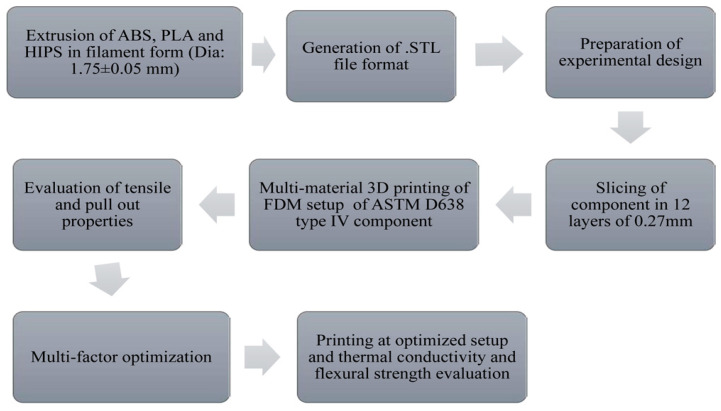
Workflow in 3D printing of MMAM parts [89].

García-Collado et al. [16] extensively reviewed polymer-based MMAM, listing the major applications and typical properties of interest. Polymers, including polymer–matrix composites like epoxy resins and fiberglass, offer excellent stiffness, flexibility, strength, and performance. They reviewed a variety of MMAM processes, such as FFF, DED, and vat photopolymerization, which use polymers such as elastomers, acrylonitrile butadiene styrene (ABS), polylactic acid (PLA), acrylic styrene acrylonitrile, nylon, vinyl ester, continuous fiber reinforcements, matrix polymers, and carbon fibers. These materials offer excellent properties, including an improved ultimate tensile strength, flexural strength, fracture toughness, biodegradability, recyclability, low density (and, thus, reduced prices), high mechanical performance, and long fatigue life. Bartlett et al. [85] tried constructing a functionally graded soft robot using the PolyJet 3D printing process. They used polymers such as elastomers and thermosets as raw materials. After a proper analysis, they concluded that the printed component showed desired variations in stiffness. An aluminum plate was joined to a polymer directly over it using fused deposition modeling (FDM) and metal extrusion processes by Falck et al. [86]. The materials used were aluminum 2024-T3, PA6, ABS, and continuous carbon fiber-polyamides. They concluded that the specimens thus obtained were sufficiently strong and that good bonding between the parent metal and the deposited polymer was obtained. The fused deposition modeling of ABS P400 material was performed by Ahn et al. [87]. Using a design-of-experiment approach, they obtained the relationship between process parameters such as the air gap, bead width, raster positioning, and tensile and compressive strengths. The results of directionally manufactured specimens were then compared with those of injection-molded FDM specimens. They deduced that the FDM components have anisotropic properties, so the strength of a particular area depends on the raster angle. Rohde et al. [88] examined the shear characteristics of 3D-printed ABS and polycarbonate components. According to them, build and raster adjustments deeply affect the shear properties of MMAM parts, varying from printer to printer. Kumar et al. [89] studied recycled ABS, PLA, and high-impact polystyrene thermoplastics for AM. The flow of the procedures followed by them can be seen in Figure 14. After a series of continuous test procedures, they noted a high Young’s modulus, enhanced flexural strength, and better thermal performance of multi-material parts compared to those of single-material specimens.

All these research works on thermoplastic-based AM clearly show that incorporating polymers with other materials to print end products has provided great advantages to the MMAM community regarding performance, durability, and adaptability.

**Figure 15:**
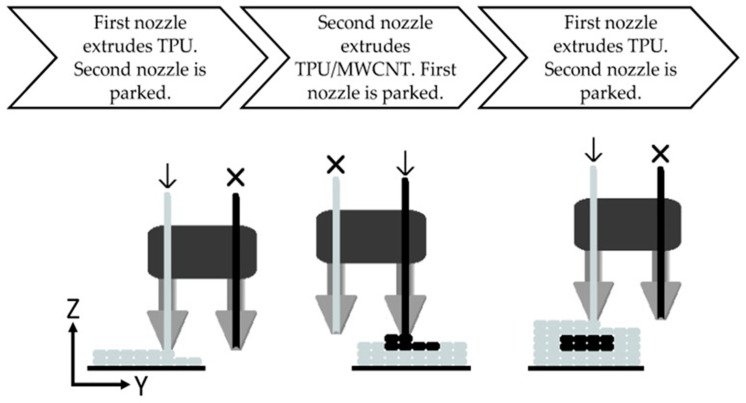
Fabrication process of multi-material strain sensors [90]. The arrows in the Figure signify that the nozzle is active and depositing while the cross mark represents an inactive nozzle.

Composite materials, on the other hand, are also a feasible option as basic materials in AM. Composites comprise two or more constituent elements, which do not dissolve into each other, with an interface being visible. Two basic forms of materials form them: one is the matrix, and the other is the reinforcement. The matrix (a polymer, a metal, or a ceramic) binds the reinforcement, shields the reinforcement from mechanical and chemical attacks, and gives the material a shape and surface finish. Reinforcements are embedded into the matrix, usually in flakes or fibers. They suffer from the entire load and give strength to the whole material. Since thermoplastics and polymers have less interfacial bonding and are sensitive to radiation and moisture, they are gradually replaced with composites, which offer excellent properties such as a high temperature and chemical resistance, high electrical and thermal conductivity, high fatigue endurance, low density, and better performance. The composite material’s strength depends on the matrix material’s length, orientation, shape, and bonding capacity and the reinforcing phase’s bonding capacity and fiber orientation. With less complex production methods such as hand layup, compression molding, pultrusion, and infiltration, these materials are now being widely used in place of conventional materials, including in marine and sports equipment, electrical transmission, aircraft, medical implants, smart materials, construction, and high-temperature applications such as rocket nozzles and gas turbines. We also find the significant use of composites in present-day AM, as in the case of Christ et al. [90], who researched the fabrication of multi-material sensors using carbon nanotube-based thermoplastic polyurethane nanocomposites by the FFF process, as illustrated in Figure 15. After repeated strain load testing, it was confirmed that the used composites showed excellent piezoresistive responses and that the obtained sensors can be employed in soft robotics and wearable electronics. In 2014, Tekinalp et al. [91] also investigated short fiber-reinforced acrylonitrile butadiene styrene composites as feedstock materials for AM processes. The built components showed an excellent tensile strength and increased modulus compared to conventional compression-molded composite blends.

#### 4.2.2. Metal–Ceramic-Based MMAM

**Figure 16:**
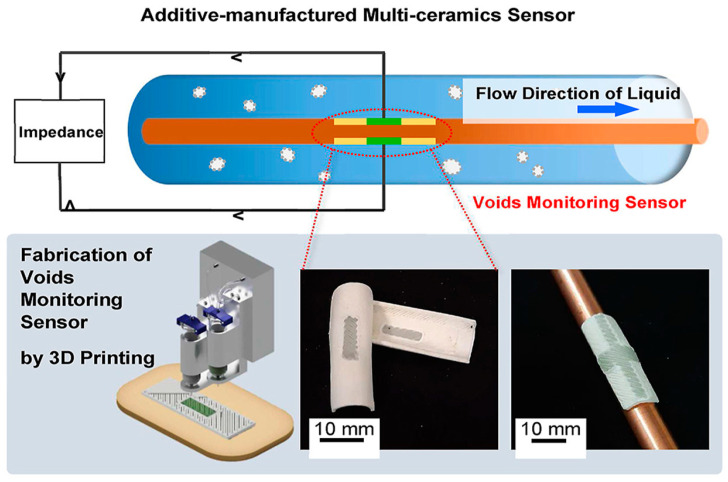
Multi-ceramic sensors [92].

**Figure 17:**
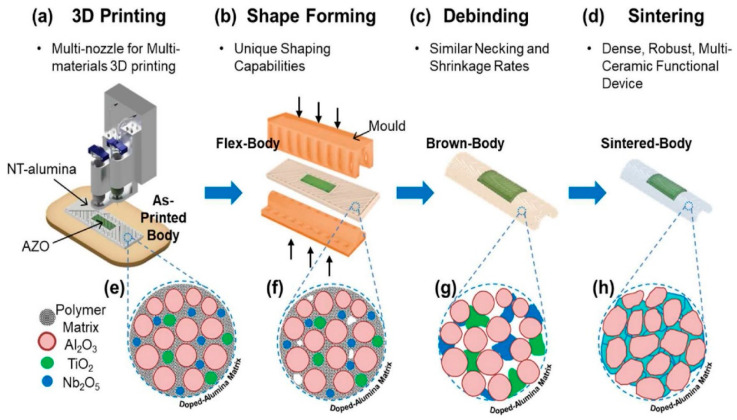
(**a–d**) Fabrication process of multi-ceramic sensors, (**e**) schematic representation of doped-alumina matrix consisting of alumina, titanium dioxide and niobium oxide powders in a binder solution, (**f**) porous ceramic green-body due to evaporation of binder solution at 150 °C, (**g**,**h**) the titanium dioxide and niobium oxide additive solution becoming a liquid state at higher temperatures [92].

Since AM came into existence, metals have been the most widely used materials as feedstock during 3D printing. Incorporating ceramics into metals is performed to improve the overall strength, fatigue endurance, wear resistance, and hardness and to improve the magnetic, thermal, and electrical characteristics. Metal–ceramic composites have been extensively used in aerospace and electronics. However, combining the two types of materials has been difficult, as both (metals and ceramics) have various melting temperatures [3]. Several researchers have worked on combining them to build products with superior qualities. Recently, a multi-ceramic sensor was developed by Zhang et al. [92] to obtain impeccable electrical conductivity and thermal insulation. The fabrication approach can be seen in Figure 16 and Figure 17. Alumina has been used as an electrically insulating material that provides mechanical strength, while alumina-doped zinc oxide has been used as an electrically conductive material. The multifunctional multi-ceramic structures thus obtained can withstand high temperatures and pressures.

In 2010, Das et al. [93] used a laser-engineered net shaping (LENS) process to give titanium metal an SiC-particle-reinforced coating to improve its wear resistance. They examined the effects of input parameters such as the laser power and speed of laser scanning on the grain morphology and crystallography, as well as the wear resistance of the coating formed. Using a metal matrix in composites has also become prominent, and metal matrix composites (MMCs) offer excellent properties such as wear resistance, conductivity, strength, and mechanical rigidity [3]. For example, using the LENS process, Balla et al. [94] fabricated a fully dense compositionally graded yttria-stabilized zirconia coating on stainless steel with a substantially improved hardness ratio. In 2010, Zheng et al. [95] also fabricated a cladding of Al + SiC powders on AZN1D magnesium alloy. After scanning electron microscopy and X-ray diffraction analysis of the cladding, it was found that, along with excellent bonding with the substrate alloy, the cladding also greatly improved the magnesium alloy’s surface hardness and wear resistance. Stainless steel (SS316) was also coated with a boron nitride reinforcement using laser processing by Heer et al. [96]. After a series of tests, it was concluded that the surface properties, such as the hardness and wear resistance, of the metal–ceramic composite were greatly affected by the addition of the reinforcement layer.

#### 4.2.3. Metal Alloy-Based MMAM

**Figure 18:**
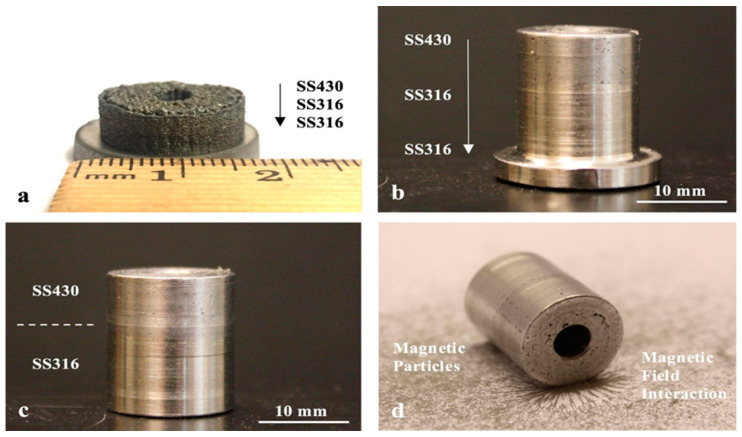
(**a**) After-LENS deposited structure; (**b**) computer numerical control (CNC)-machined sample with the base; (**c**) final machined structure from a CNC machine; (**d**) validation of improved magnetic properties [100].

**Figure 19:**
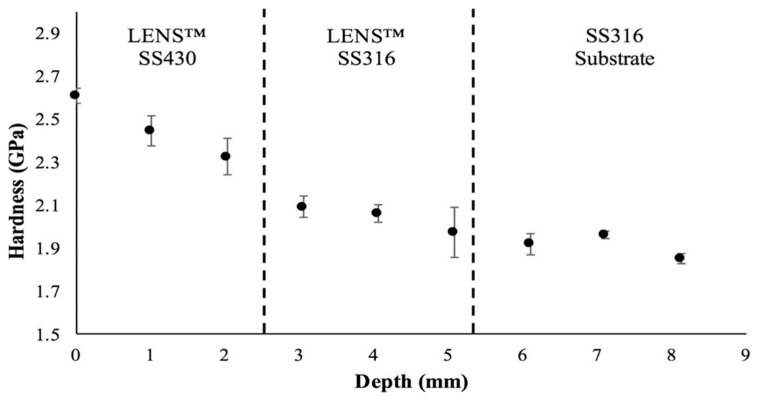
Plot of the hardness (in GPa) along the depth (in mm) of the substrate after LENS [100].

Metal additive manufacturing (MAM) is the most used AM method for producing end-use products in the aerospace (rocket nozzles, fuel injector nozzles), automotive, biomedical (tissue engineering), and construction industries and in many other applications. Metals such as titanium, aluminum, copper, and stainless steel have been used in 3D printing [2]. Combining metals and alloys with AM processes gives designers great power to influence and tailor the properties of the end-use product according to their needs [3]. The mechanical behavior of an element is primarily affected by its texture, which further depends on process parameters such as the beam speed, beam power, and heat source during MAM [97]. To improve the wear resistance of a titanium alloy, Wang and Wang [98] used a laser cladding process to coat the substrate titanium alloy with a Ti2Ni3Si-reinforced intermetallic. After dry sliding wear tests, it was found that these alloys with the metallic coating have improved wear resistance and hardness properties. As stated in the critical review of Hofmann et al. [99], combining IN625 and SS304 by the DED AM process results in an improved surface finish and improved hardening effects of the base metal. For a specific area-based functionality, Heer and Bandyopadhyay [100] used the LENS process to combine nonmagnetic austenitic stainless steel 316 (SS316) with magnetic ferritic stainless steel 430 (SS430) in one structure, which decreased its porosity and shifted the magnetic properties to a moderate level. The machined parts are shown in Figure 18, and the variation in the hardness level along the depth of the structure is plotted in Figure 19.

**Figure 20:**
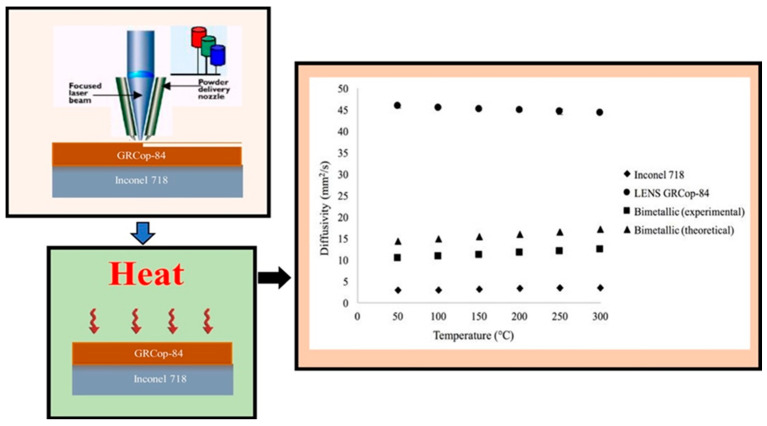
LENS process used to fabricate an Inconel 718-GRCop-84 bimetal; demonstration of the improved thermal diffusivity of the bi-metallic structure printed [102].

Recently, Groden et al. [101] fabricated bi-metallic structures combining Inconel 718 and CoCrMo using a DED-based MAM process to combine individual characteristics such as wear and fatigue resistance and high-temperature oxidation. The resultant bi-metallic component was free of cracks, porosity, or interfacial phase formation. Thus, the authors confirmed that a stable bimetal was formed, which can be used to fabricate other structures to impart corrosion/wear resistance properties to them. Onuike et al. [102] also formed a bi-metallic component from Inconel 718 and the copper alloy GRCop-84 using the LENS process. The so-formed bimetal showed an improved thermal diffusivity compared to pure Inconel 718 by approximately 250%. The schematic diagrams of the processes involved and the thermal diffusivity plot validating its improved value for the bimetal formed are shown in Figure 20. Structures such as these pave the way for multi-material AM, particularly for high-temperature applications such as aerospace applications.

#### 4.2.4. Biomaterials

MMAM has successfully created in situ alloys, FGMs/CGMs, and bi-metallic structures for biomedical applications, such as titanium alloys for implants in the orthopedic sector. Mostly, Fe-based, Ti-based, and Mg-based alloys are being used as multifunctional biomaterials. The metal extrusion MMAM process is reported to be extremely efficient in manufacturing metallic biomaterials [4]. Zadpoor and Malda [103] examined the use of AM in the healthcare industry to produce prosthetics, drug delivery devices, implants, orthotics, microfluidic devices, and medical instruments. Using finite element analysis, Campoli et al. [104] studied the mechanical behavior of metallic biomaterials produced by the SLM method. It has been reported that the irregularities formed during the printing process affect the mechanical strength of titanium biomaterials. The cross-section of Ti6Al4V implants built by 3D printing was evaluated after six months of use using microscopy techniques by Shah et al. [105]. In 2011, Gu et al. [106] manufactured TiC/Ti5Si3 in situ biomaterials using PBF (SLM process), which were wear-resistant and hard. In situ TiB/Ti-6Al-4V nanocomposites for biomedical applications with increased microhardness were also synthesized using the SLM process [107]. Shuai et al. [108] examined the effectiveness of the Nb-induced intermetallic phase in the Mg alloy Mg-5.6Zn-0.5Zr (ZK60) in improving its corrosion resistance properties for further usage in bone implants. After considerable tests, ZK60-3.6Nd was a reliable option for biodegradable bone implants. Samuel et al. [109] presented their studies on LENS-formed Ti-Nb-Zr-Ta biocompatible alloys by concluding that the corrosion resistance of the formed alloy is very much improved, and in vitro examinations were also performed, which yielded favorable and positive results. Using inkjet-based AM methods, Chou et al. [110] processed Fe-Mn alloy-based biomaterials for bone scaffold materials, which exhibited improved mechanical behavior and corrosion resistance and were a better option for craniofacial biomedical uses. Furthermore, they also worked on developing new biodegradable Fe-Mn-Ca/Mg alloys printed by binder jetting methods for similar applications. They concluded that these alloys were better candidates with increased cytocompatibility [111].

### 4.3. Capabilities of MMAM

MMAM allows the manufacturer to appropriately choose the most suitable materials to build the specific components, keeping in mind their application, cost, and availability. MMAM has also successfully fabricated some special materials and structures that have been extremely difficult to produce with conventional manufacturing processes and are widely used in the modern era. Thus, MMAM is creating a new paradigm in the design of smart materials. In the following section, such special applications of MMAM are discussed in detail.

#### 4.3.1. Bi-Metallic Components

**Figure 21:**
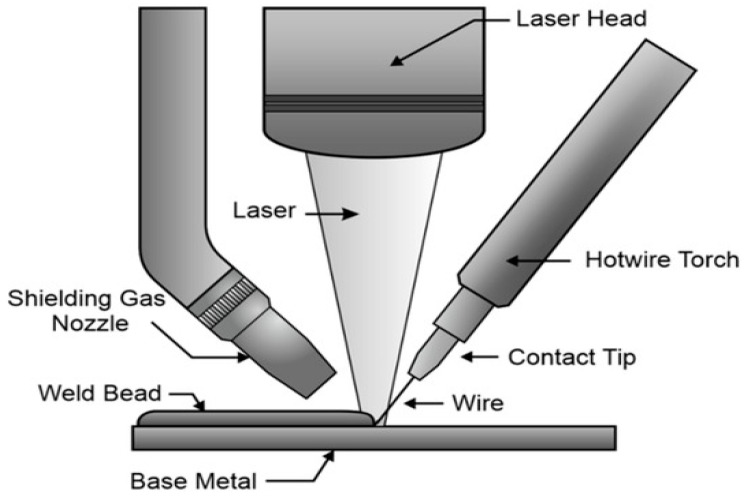
Laser-hot wire AM process [114].

Combining two metals to form objects with the superior characteristics of both metals for special applications is called bimetal fabrication. Due to the ease of operation, AM has been mostly used for bi-metallic object manufacturing. Zhang et al. [112] recently developed dissimilar multi-material structures of SS316L-D22-Cu (pure copper on SS316L with interlayers of Deloro-22) using laser DED. They concluded that the interfacial bonding was strong, and no cracking was observed. Additionally, significant increases in thermal diffusivity and thermal conductivity were measured. Yusuf et al. [113] used laser-based PBF methods to combine 316L steel and Inconel 718. They mentioned that a good microhardness value and a great metallurgical bond are obtained at the interface. The thermal expansion properties of bi-metallic Invar M93 beads on an A36 steel base fabricated using laser-based wire AM were investigated by Arbogast et al. [114]. They deduced that the multi-material AM of Invar considerably decreased the coefficient of thermal expansion of the entire system. A schematic diagram of the AM process used to fabricate Invar–steel bimetals is shown in Figure 21.

To manufacture titanium-based alloy and nickel-based alloy bi-metallic components (TC4-IN718), which are widely used in the aerospace industry due to their excellent characteristics, tantalum–copper interlayers were utilized by Wang et al. [115] to prevent the formation of Ti-Ni and Ti-Cu compounds at the interface and thus form a good bond at the interface. The formed bimetal showed an ultimate tensile strength of 370 N/mm^2^. Despite its limited use due to the increased cooling time, the DED process used injection molding by Bennett et al. [116] to deposit 17-4 PH stainless steel onto copper molds. A reduced lead time and an increased mold life were reported, thus indicating the potential of the multi-material injection molding process. In more recent work with the utilization of WAAM, the bi-metallic pairs LCS-SS [117] and CRS-SS [118,119] have been successfully fabricated.

#### 4.3.2. Functionally/Compositionally Graded Materials

**Figure 22:**
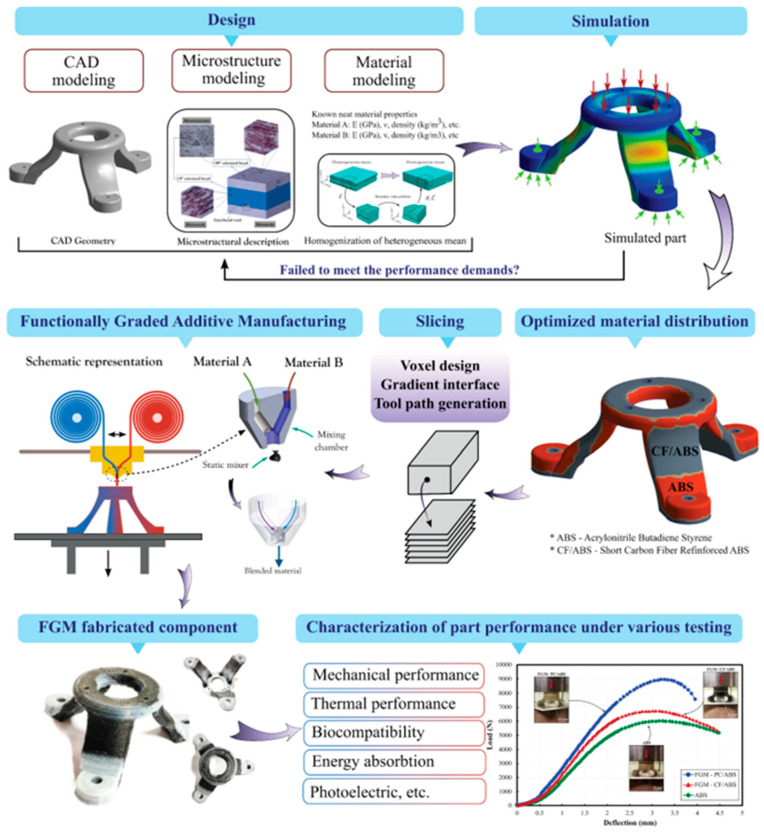
Functionally graded additive manufacturing process [2].

Functionally graded materials are components in which the composition of the material varies along the cross-section of the fabricated component to meet its specific functional requirements. This is often achieved by altering the process parameters or using special equipment. FGMs have extensive usage in aerospace, automobile, biomedical, and defense areas. Machine learning algorithms were used by Rankouhi et al. [120] to fabricate compositionally graded SS316L-Cu multi-material structures. Using the WAAM process, FGMs were built for applications in the marine sector to fulfill the need for a corrosion-resistant material. Sharma et al. [121] manufactured tailor-made functionally graded composites through friction stir additive manufacturing. The construction laser additive direct (DED-CLAD) process was utilized to manufacture a Ti6Al4V-molybdenum-based FGM for biomedical applications [122]. Savitha et al. [123] tested a CGM built with SS316 and IN625 materials. The excellent interfacial bonding of the CGM was proven by a series of chemical and mechanical tests. Tan et al. [124] developed a steel-copper FGM using LPBF, which, upon several mechanical tests, displayed high interfacial strength. Melzer et al. [125] utilized the Blown Powder Directed Energy Deposition system to fabricate a functionally graded composite (FGC) consisting of stainless steel 316L and Inconel 718. They investigated the mechanical properties of the FGC within single layers as well as over layers using tensile and fracture toughness tests. It was observed that the interfacial crack propagation mechanism is dependent on the type of transition between material interfacial layers. Along with numerous advantages, FGMs have certain limitations, such as the lack of material composition variation in some AM systems, and the capacity to use only one material per printing hinders FGMs from overtaking conventional materials [126]. Figure 22 depicts the processing route employed for producing FGMs.

#### 4.3.3. Alloy Design

**Figure 23:**
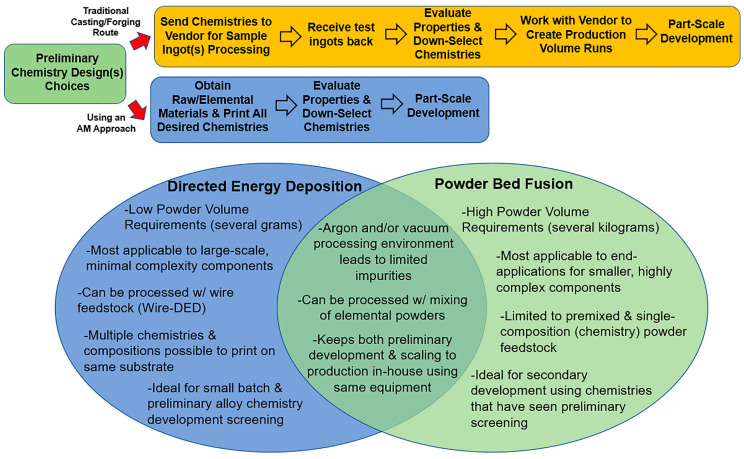
Comparison of traditional methodologies and DED and PBF processes for alloy design [127].

AM has certainly created a revolution in the present times, with numerous merits over conventional processing methods. Using multiple materials (MMAM) further provides an additional advantage by incorporating two or more materials in a single component during printing to maximize its performance and durability and minimize the assembly cost. Owing to these advantages, novel alloys with application-specific compositions of different kinds of metals have started to be designed and evaluated using AM. Earlier, alloy design was limited to tedious traditional casting methods. However, introducing AM processes, namely, DED and PBF, into in situ alloying has reduced the overall cost, improved the microstructure and, thus, the strength, and reduced the lead time. A detailed comparison of traditional and AM processes for alloy design is illustrated in Figure 23. Various alloys, including titanium alloys, nickel-based alloys, and aluminum alloys, have been developed using AM processes for applications in areas such as the aerospace and biomedical industries [127].

Martin et al. [128] used the PBF process to print aluminum alloy 6061/7075 with Zr particles to reduce the porosity and, thus, fracture. Using a niobium reinforcing phase, an Inconel (Ni+ Cr) alloy was fabricated using the DED process by Vecchio et al. [129] to increase the hardness of the superalloy. Mitra et al. [130] manufactured a tantalum–titanium-based alloy to increase the biocompatibility of titanium for applications in the biomedical and orthopedic industries. Twin WAAM was utilized by Yang et al. [131] to combine various elements to build alloys, such as the Ti-6Al-7Nb alloy with improved mechanical behavior and grain structure. Dong et al. [132] also used WAAM to build a Cu-Al alloy, resulting in improved strength, ductility, and the desired chemical state.

### 4.4. Scale of MMAM

**Figure 24:**
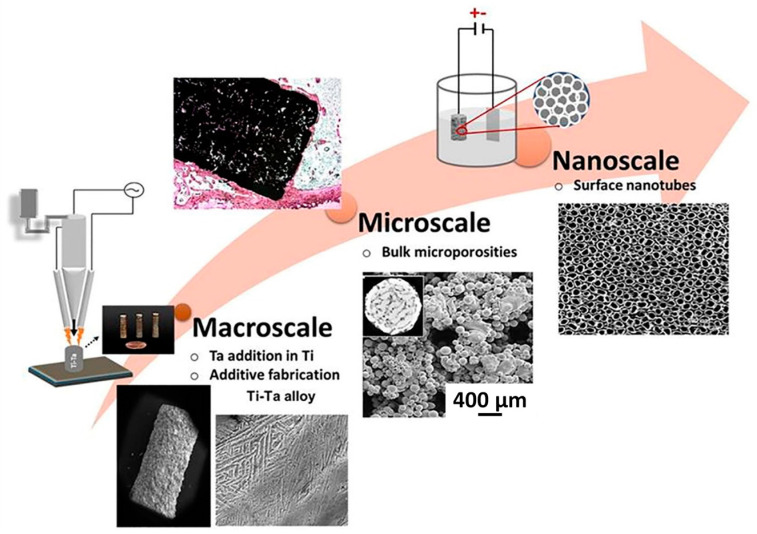
Multiplicity in scale in MMAM [127].

The possibilities of MMAM range from microscale production to large-scale/construction-scale production. Figure 24 shows the MMAM multiplicity in scale, where Ta was incorporated into Ti using DED, which combined in situ alloying and surface modification. In large-scale construction, the advent of MMAM can lead to a paradigm shift in how components and buildings are designed, constructed, and manufactured. Implementing MMAM in large-scale production enables the introduction of assembly-free construction [21]. The flexibility of MMAM with regard to the scale of production provides several benefits in comparison to single-material AM, including but not limited to functional integration across distinct and/or different materials [133], a reduction in the part count [21,133], transitional grading across materials [114], the ability to adjust properties across the volume of the product [134], and 4D printing. Although only limited research results and examples of practical implementation are available for large-scale MMAM, the initial prototype productions are very promising and pave the way for further developments and the introduction of new concepts in the realm of large-scale MMAM. Large-scale MMAM has tremendous potential in the aerospace, construction, and architecture sectors, where weight and cost are of prime concern. A recent report on large-scale MMAM suggested that a multi-material sandwich structure would reduce the weight and costs by approximately 5% and 45%, respectively [135].

Mesoscale structures with multiple materials are highly efficient and capable of adapting to real-world environmental changes. With the rapid development of MMAM and the capabilities of associated AM techniques, the production of multi-material systems with user-defined mesoscale material distributions has become a reality. This provides significant freedom of design at characteristic length scales between the macroscale geometry and microstructures [136]. As MMAM provides unique pathways for implementing an arbitrary mesoscale material distribution, there is a growing need to develop new design frameworks to optimize the mesoscale material distribution.

Almost all available microscale AM processes for single-material deposition can be modified or scaled for multi-material capabilities. However, much effort is needed to develop an in-depth understanding of the behavior and compatibility of different materials intended for functional application at microscales [137]. Although well-developed system designs, deposition strategies, an understanding of material consolidation, etc. are available for single-material AM at the microscale, the same knowledge is lacking for MMAM.

### 4.5. Structural Aspects of MMAM

**Figure 25:**
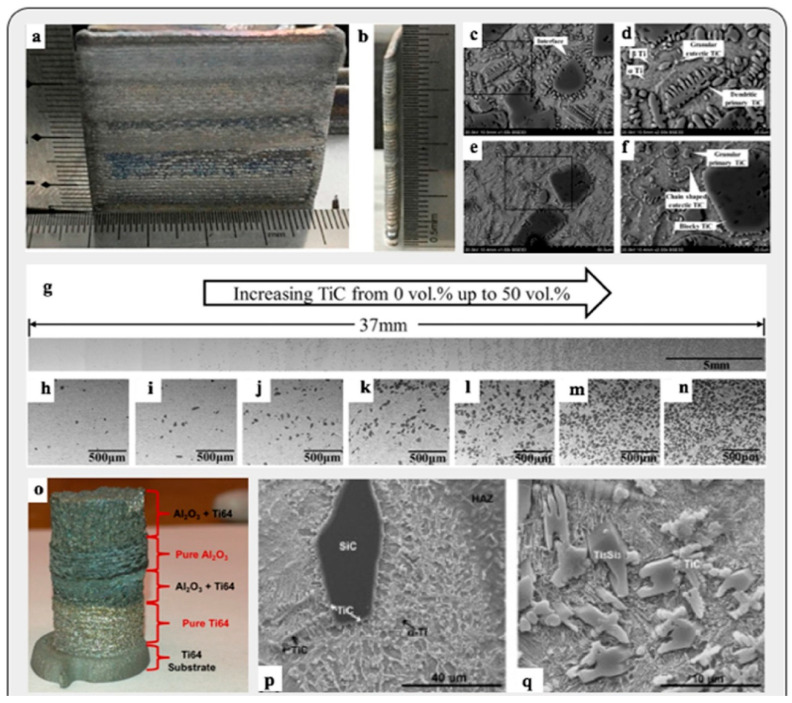
Multiplicity in structure in MMAM, (**a**–**f**) addition of TiC particles during wire feed processing, (**g**–**n**) particle morphology with respect to increasing particle addition, (**o**) novel Ti6Al4V + Al_2_O_3_ CGM, and (**p**,**q**) TiC reaction product in a SiC reinforced titanium coating [3].

**Figure 26:**
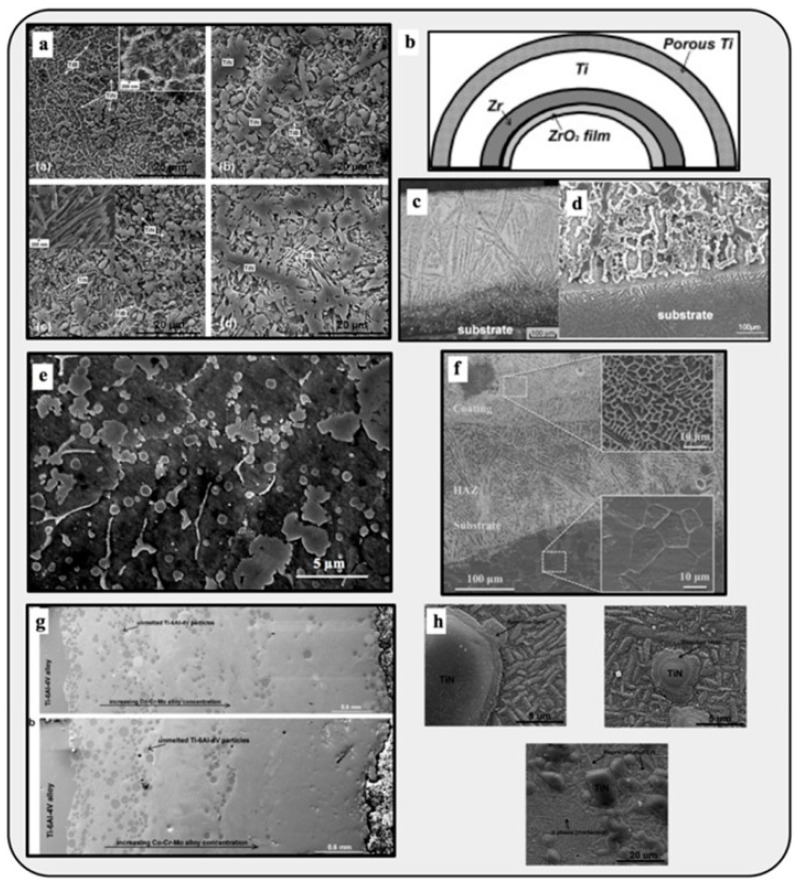
Microstructures and phases in metal-based MMAM: (**a**) nitrides in BN-reinforced Ti6Al4V, (**b**) design for MMAM, (**c**,**d**) intermetallic formation at the interface of a Ti3Ni2Si-reinforced composite, (**e**) Cu alloy on IN718, (**f**) interface of an SS316 + BN composite coating, (**g**) Ti6Al4V with an increasing amount of CoCrMo [74], and (**h**) reaction layers around TiN formation in TiN-reinforced Ti6Al4V [3].

MMAM provides the unique ability to produce parts with specific properties in desired areas and allows for property and corresponding structure variation in a single operation. MMAM can provide better control over the material properties, provided that proper design choices are implemented, thus allowing for the production of a myriad of exotic and never-before-seen structures [136]. Figure 25 shows the various metal–ceramic structures and microstructures produced using MMAM techniques. Figure 25a–f depict the microstructures produced when TiC particles were added in the wire DED process, Figure 25g–n depict the structure with an increasing particle addition, Figure 25o depicts a Ti_6_Al_4_V + Al_2_O_3_ compositionally graded structure, and Figure 25p,q depict the TiC reaction product in an SiC-reinforced titanium coating. Figure 26 highlights the distinctive microstructures and phases produced during metal-based MMAM.

### 4.6. Material Interaction and Related Issues in MMAM

The aspect of material interaction plays a distinctive role in the formation of strong defect-free components in MMAM and is an ongoing research interest. Even though MMAM is capable of fabricating FGMs, bi-metals, and other exotic structures, the interaction of multiple materials requires a deeper understanding of several phenomena like the formation of brittle intermetallics and low-melting eutectics, inadequate fusion at the multi-material interface(s), elemental segregation, crack formation, etc. With the increased demand for complex multi-material structures with features like property gradation and localized property manipulation, the interfacial characterization and understanding of the process–structure–property relationships are crucial for the fabricated parts’ performance and integrity. The material combination and the orientation of the material interfaces, in addition to the processing parameters, have a significant impact on the interfacial strength of the multi-material component [44,117]. To alleviate the issue of the reduced ductility of intermetallic phases in multi-material structures, Chen et al. [137] introduced a third metal element into the MMAM process, which led to an increase in the ductility of the intermetallic phase. More recently, Banaee et al. [119], in their study on the WAAM of CRS-SS bi-metallic walls, found that the difference in material fluidity has a drastic effect on the interfacial fusion. They advocated that, unlike for single-material deposition, for multi-material deposition, a proper combination of process parameters for each material, the sequence of the deposition of the materials, and the overlapping distance, together, control the resulting fusion state (or lack of it) in the multi-material interface. Despite substantial advances made in metal–ceramic MMAM, the highly disparate material properties and resulting high thermal stress from fast solidification processes make metal–ceramic structures vulnerable to cracking. In the case of metal-polymer MMAM, one of the most critical issues is the prevention of the gasification and disintegration of polymer powders with a low melting point. Amoabeng and Velankar [138] suggested the use of metal and polymer powders with relatively similar melting points to overcome this limitation.

## 5. Conclusions and Outlook

This paper covers various multiplicity aspects of MMAM, including the process, material, scale, and structure, and also highlights advantages, challenges, applications, and future research directions in MMAM. The review indicates that the MMAM technologies, owing to their inherent multiplicity aspects, have the potential to become a key means for the next generation of manufacturing technologies. In recent years, MMAM has seen increased adoption for the fabrication of complex metallic and non-metallic structures. For multi-material applications that are difficult to realize with conventional manufacturing processes, MMAM can act as a viable and energy-efficient solution. The major conclusions of this review are:

- MMAM presents solutions that directly impact the efficient use of vital resources such as materials, energy, and time, resulting in a shorter process chain. For instance, bi-metallic pipe bends, currently produced through explosive cladding followed by rolling and limited to standard geometries, can benefit from the design and manufacturing freedom offered by multi-material AM.
- All the existing processes for single-material AM are also applicable to MMAM. However, selecting a process version becomes highly material-specific in the case of MMAM. Therefore, a precise understanding of the process–structure–property relationship and the seamless implementation of the additive–subtractive process chain are essential for the success of MMAM.
- Material interaction in MMAM is intricately related to deposition processes and operating conditions, making the MMAM process highly interactive. Transitioning from single- to multi-material requires extensive experimentation since solutions that work for individual process–material combinations may not apply to their multi-material counterparts. Developing new products with MMAM remains time-consuming and necessitates significant financial investments.
- Properly joining dissimilar material classes remains one of the most significant challenges in current MMAM technologies. Ongoing AM procedures aim to tackle this issue through the development of customized feedstock like filler wires and powders. There is a pressing need for more efficient and sustainable solutions, such as in situ alloy deposition employing MMAM.
- Before the full-scale industrial implementation of MMAM can be realized, several issues need to be addressed, including numerical simulations of complex phenomena, the absence of standardized processing parameters for machines from different suppliers across material classes, and a lack of literature for accurate cost estimations.
- The advent of Additive–Subtractive Multi-Material Additive Manufacturing (ASMMAM) is expected to enable the quick repair of cracks and surface defects, a feat that was previously challenging with available technologies. Repairing and cladding parts through ASMMAM opens exciting opportunities for developing new applications and components.

Although this review covers various multiplicity aspects of MMAM, several topics including but not limited to topology optimization, process modeling, design methodologies and software, mass transfer phenomena, in-machine surface metrology, post-processing, advanced monitoring and sensor fusion for obtaining in situ information of phase formations and microstructure evolution, path planning algorithms, quality control, and material-dependent manufacturing framework conditions, which are crucial for the development of MMAM, have been excluded to limit the scope of the review. The creation of a knowledge base through the systematic study of geometric features and geometry-related quality requirements that can enable the fabrication of large MMAM components fall outside the purview of this review article.

## Figures and Tables

**Table 1:** Overview of processes for MMAM (adapted from [47]).

Categories	Technologies	Printed Ink	Power Source	Strengths/Downsides
Material Extrusion	Fused Deposition Modeling (FDM)	Thermoplastics, Ceramic slurries, Metal pastes	Thermal Energy	▪ Inexpensive extrusion machine ▪ Multi-material printing ▪ Limited part resolution ▪ Poor surface finish
	Contour Crafting			
PBF	Selective Laser Sintering (SLS)	Polyamides/Polymer	High-powered Laser Beam	▪ High accuracy and details ▪ Fully dense parts ▪ High specific strength and stiffness ▪ Powder handling and recycling ▪ Support and anchor structure ▪ Fully dense parts ▪ High specific strength and stiffness
	Direct Metal Laser Sintering (DMLS)	Atomized metal powder (17−4 PH stainless steel, cobalt chromium, titanium Ti6Al-4V), ceramic powder	Electron Beam	
	Selective Laser Melting (SLM)			
	Electron Beam Melting (EBM)			
Vat Photopolymerization	Stereolithography (SLA)	Photopolymer, Ceramics (alumina, zirconia, PZT)	Ultraviolet Laser	▪ High building speed ▪ Good part resolution ▪ Overcuring, scanned line shape. ▪ High cost for supplies and materials
Material Jetting	Polyjet/Inkjet Printing	Photopolymer, Wax	Thermal Energy/Photocuring	▪ Multi-material printing ▪ High surface finish ▪ Low-strength material
Binder Jetting	Indirect Inkjet Printing (Binder 3DP)	Polymer powder (Plaster, Resin), Ceramic powder, Metal powder	Thermal Energy	▪ Full-color objects printing ▪ Require infiltration during post-processing ▪ Wide material selection ▪ High porosities on finished parts
Sheet Lamination	Laminated Object Manufacturing (LOM)	Plastic Film, Metallic Sheet, Ceramic Tape	Laser Beam	▪ High surface finish ▪ Low material, machine, and process costs ▪ Decubing issues
Directed Energy Deposition	Laser Engineered Net Shaping (LENS), Electronic Beam Welding (EBW)	Molten Metal Powder	Laser Beam	▪ Repair of damaged/worn parts ▪ Functionally graded material printing ▪ Require a post-processing machine
